# *Notes from the Field:* Primary Amebic Meningoencephalitis Associated with Nasal Irrigation Using Water from a Recreational Vehicle — Texas, 2024

**DOI:** 10.15585/mmwr.mm7419a4

**Published:** 2025-05-29

**Authors:** Olivia A. Smith, Whitney Tillman, Jantel B. Lewis, Stephen White, Mia Mattioli, Julia Haston, Megan Dorris, Amy Kahler, Alexis Roundtree, Ibne Karim Ali, Shantanu Roy, Taylor Yakubik, Lauren Sisco, Jasen Kunz

**Affiliations:** ^1^Emerging and Acute Infectious Disease Unit, Texas Department of State Health Services; ^2^Division of Foodborne, Waterborne, and Environmental Diseases, National Center for Emerging and Zoonotic Infectious Diseases, CDC; ^3^Department of Infectious Diseases, Baylor Scott & White Medical Center, College Station, Texas.

**Summary**

**What is already known about this topic?**

Primary amebic meningoencephalitis (PAM) is a rare, often fatal brain infection caused by the free-living ameba *Naegleria fowleri*. Using tap water for nasal irrigation is a risk factor for PAM.

**What is added by this report?**

A fatal case of PAM occurred in an otherwise healthy adult woman who used tap water obtained from her recreational vehicle (RV) in a nasal irrigation device. Although *N.* *fowleri *was not isolated from the RV water supply, the water was found to be inadequately disinfected.

**What are the implications for public health practice?**

This case highlights the importance of following recommended nasal irrigation practices. Improperly maintained RV water systems can be a source of waterborne disease, including PAM.

Primary amebic meningoencephalitis (PAM) is a rare, often fatal brain infection caused by the free-living ameba, *Naegleria fowleri* (*1*). Infection is typically associated with recreational water activities; however, using tap water when performing nasal irrigation is also a risk factor for PAM (*2*–*4*). Improperly maintained municipal water and recreational vehicle (RV) water systems can be a source of waterborne disease; CDC recommends the use of distilled, sterile, or boiled and cooled tap water for nasal irrigation.* Household safe water practices can help prevent waterborne illness associated with RV water systems. This report describes a fatal case of *N. fowleri* infection associated with improper use of a nasal irrigation device with suspected contaminated tap water from an RV.

## Investigation and Outcomes

### Case Identification

A previously healthy woman aged 71 years developed severe neurologic symptoms, including fever, headache, and altered mental status within 4 days of using a nasal irrigation device filled with tap water from an RV’s water system at a campground in Texas. Despite medical treatment for a suspected PAM infection, the patient developed seizures and subsequently died 8 days after symptom onset. Laboratory testing at CDC confirmed the presence of *N. fowleri* in the patient’s cerebrospinal fluid.

### Identification of Potential Sources of Contamination

An epidemiologic investigation conducted by the Texas Department of State Health Services included a review of the patient’s medical and exposure history. The patient had no recreational exposure to fresh water; however, she had reportedly performed nasal irrigation on several occasions using nonboiled water from the RV potable water faucet during the 4 days before illness onset. This practice suggested two water sources of concern. The first was the RV’s potable water tank, which flowed directly to the faucets and shower when a municipal water connection was unavailable. The tank had been filled with water collected on an unknown date before the patient’s purchase of the RV 3 months earlier. The second potential source of contamination was the municipal water system, which was connected by a hose and water filter to the RV potable water system, bypassing the tank, at the time the patient used it for nasal irrigation.

### Environmental Sampling

To evaluate these water sources, investigators collected 12 environmental samples. These samples included the patient’s nasal irrigation squirt bottle; 1 liter of water from the RV water heater; swabs from the RV shower head and kitchen and bathroom sink faucets; one large volume (approximately 4 gal [15 L]) ultrafiltered water sample and one swab of the RV potable water tank; one large volume (approximately 26 gal [100 L]) ultrafiltered water sample and one swab from the campsite municipal water where the RV connected; the RV external water filter; the RV municipal connection hose; and another large volume (approximately 26 gal [100 L]) ultrafiltered water sample from a low flow (i.e., dead-end) campsite municipal water connection.

### Testing for *N. fowleri* and Water Quality

Physical and chemical water quality parameters were assessed at the time of sampling, and all samples were tested for *N. fowleri* at CDC. No *N. fowleri* DNA or viable ameba were detected in any environmental samples collected at the campsite water sources or in the RV water system. However, the total chlorine and monochloramine (i.e., disinfectant) levels in the low flow campsite municipal distribution system sample (both <0.04 mg/L) were below the minimum disinfectant residual levels recommended by the Texas Commission on Environmental Quality (≥0.50 mg/L monochloramine or total chlorine).^†^ In addition, the presence of free ammonia, lower pH (<8.5), and unequal concentrations of active disinfectant (measured as a concentration of monochloramine) and total chlorine (which represents all forms of chlorine, including less effective forms) at the campsite where the RV was connected indicated suboptimal disinfection efficacy, which might have led to biofilm growth. Biofilm can grow when water becomes stagnant or disinfectant residuals are depleted, resulting in pathogen growth. Although no test for the presence of biofilms exists, biofilms can act as a protective shield for pathogenic microorganisms, including bacteria and amebas such as *N. fowleri*, making the amebas less susceptible to disinfectant (*5*). Further, the turbidity (i.e., the cloudiness of water) measured at taps and inside the RV was significantly higher (range = 1.26–4.32 nephelometric turbidity units [NTUs])^§^ than that recommended for drinking water (<1.0 NTU), suggesting a disinfection breakdown. Insufficient disinfectant residual entering the RV and high turbidity at the point of use might have contributed to the presence of thermophilic ameba, although these were not detected in the samples tested.

## Preliminary Conclusions and Actions

Nasal irrigation using tap water remains the suspected route of exposure, given the absence of other identified nasal water exposure and the concerning quality of the campground municipal water and RV tap water at the time of sampling. Failure to isolate the organism from the samples collected might be explained by the fact that sampling occurred 23 days after the patient used the water for nasal irrigation, and the environmental conditions might have differed from those present when infection occurred. In addition, the pathogen might have been present at the time of sampling but at levels below the detection limit. Whether the municipal water system or the RV potable water tank was the source of contamination is unknown, because the tank might have contaminated the RV potable water system before connection to the campground municipal water system.

This case reinforces the potential for serious health risks associated with improper use of nasal irrigation devices, as well as the importance of maintaining RV water quality and ensuring that municipal water systems adhere to regulatory standards. Following recommended nasal irrigation practices, which include using distilled, sterilized, or boiled and cooled tap water for nasal irrigation, is critical to reducing the risk for illness. Because of this investigation, Texas public health officials and CDC waterborne disease experts collaborated to create recommendations for safer RV water usage and storage to mitigate the risk for waterborne diseases associated with RV water systems (Supplementary Figure).

## Footnotes

*
How to Safely Rinse Sinuses | *Naegleria fowleri* Infection | CDC

† Total chlorine, monochloramine, free ammonia, total ammonia, and nitrite levels were tested at the time of sampling using a Hach SL1000 portable parallel analyzer and portable colorimeter with the following results: in the water at the campsite where the RV was connected, monochloramine = 0.05 mg/L, total chlorine = 0.63 mg/L, pH = 7.74, free ammonia = 0.18 mg/L, total ammonia = 0.28 mg/L, and nitrite >0.69 mg/L. In the water at the low flow campsite (dead-end), monochloramine <0.04 mg/L, total chlorine <0.04 mg/L, pH = 7.50, free ammonia = 0.11 mg/L, total ammonia = 0.11 mg/L, and nitrite = 0.65 mg/L.

§ Turbidity was measured using a Hach 2100P turbidity meter in a CDC laboratory. The Environmental Protection Agency requires that turbidity not exceed 1 NTU in systems using conventional or direct filtration. Measured municipal campground water = 1.26 NTU at the dead-end connection and 1.54 NTU at the RV connection; RV tap water = 1.95 NTU at the bathroom sink tap and 4.32 NTU at the kitchen sink tap.
